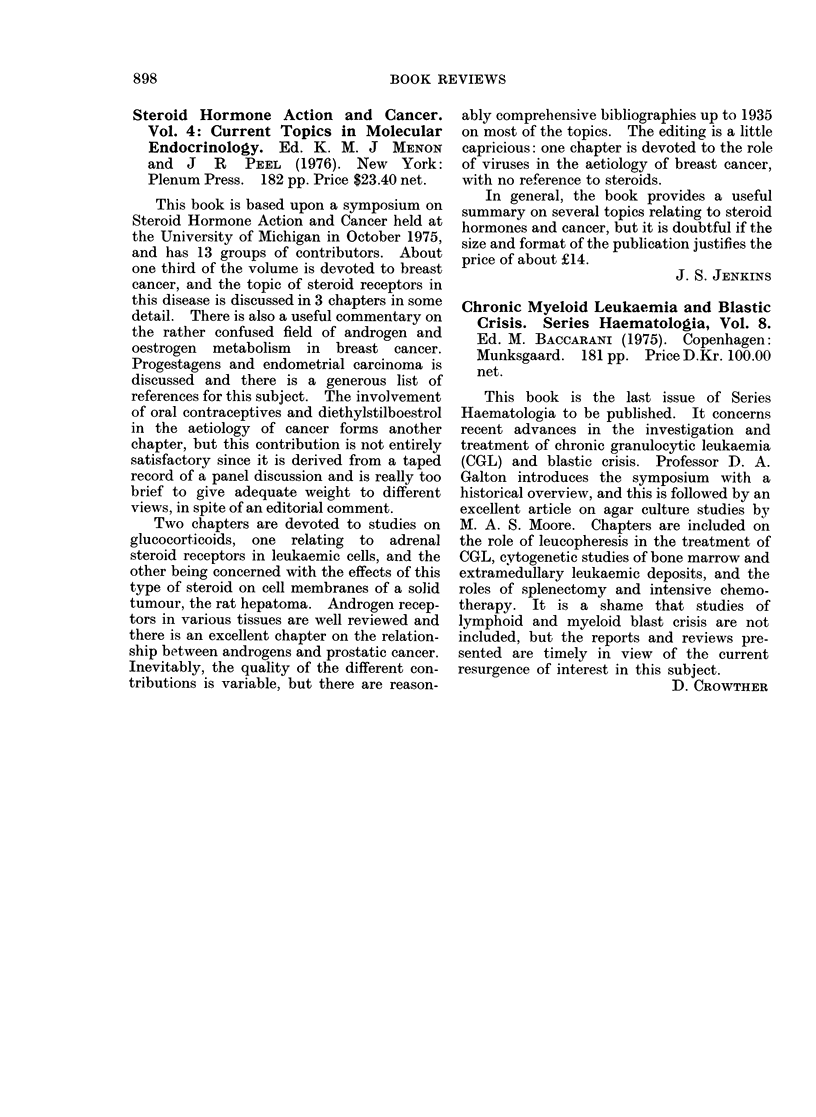# Chronic Myeloid Leukaemia and Blastic Crisis. Series Haematologia, Vol. 8

**Published:** 1977-06

**Authors:** D. Crowther


**Chronic Myeloid Leukaemia and Blastic Crisis. Series Haematologia, Vol. 8.** Ed. M. Baccarani (1975). Copenhagen: Munksgaard. 181 pp. Price D.Kr. 100.00 net.

This book is the last issue of Series Haematologia to be published. It concerns recent advances in the investigation and treatment of chronic granulocytic leukaemia (CGL) and blastic crisis. Professor D. A. Galton introduces the symposium with a historical overview, and this is followed by an excellent article on agar culture studies by M. A. S. Moore. Chapters are included on the role of leucopheresis in the treatment of CGL, cytogenetic studies of bone marrow and extramedullary leukaemic deposits, and the roles of splenectomy and intensive chemotherapy. It is a shame that studies of lymphoid and myeloid blast crisis are not included, but the reports and reviews presented are timely in view of the current resurgence of interest in this subject.

D. Crowther